# Biogenesis and Function of Multivesicular Bodies in Plant Immunity

**DOI:** 10.3389/fpls.2018.00979

**Published:** 2018-07-09

**Authors:** Xifeng Li, Hexigeduleng Bao, Zhe Wang, Mengxue Wang, Baofang Fan, Cheng Zhu, Zhixiang Chen

**Affiliations:** ^1^Department of Horticulture, Zhejiang University, Hangzhou, China; ^2^College of Life Sciences, China Jiliang University, Hangzhou, China; ^3^Department of Botany and Plant Pathology, Center for Plant Biology, Purdue University, West Lafayette, IN, United States

**Keywords:** MVBs, plant immunity, LIP5, endocytosis, endosomal trafficking

## Abstract

Multivesicular bodies (MVBs) are specialized endosomes that contain intraluminal vesicles generated from invagination and budding of the limiting membrane. In the endocytic pathway, MVBs are late endosomes whose content can be degraded through fusion with lysosomes/vacuoles or released into the extracellular space after fusion with the plasma membrane (PM). The proteins retained on the limiting membrane of MVBs are translocated to the membrane of lysosomes/vacuoles or delivered back to the PM. It has been long suspected that MVBs might fuse with the PM to form paramural bodies in plant cells, possibly leading to release of building blocks for deposition of papillae and antimicrobial molecules against invading pathogens. Over the past decade or so, major progress has been made in establishing the critical roles of MVBs and associated membrane trafficking in pathogen recognition, defense signaling, and deployment of defense-related molecules during plant immune responses. Regulatory proteins and signaling pathways associated with induced biogenesis and trafficking of MVBs during plant immune responses have also been identified and characterized. Recent successful isolation of plant extracellular vesicles and proteomic profiling of their content have provided additional support for the roles of MVBs in plant–pathogen interactions. In this review, we summarize the important progress and discuss how MVBs, particularly through routing of cellular components to different destinations, contribute to the complex network of plant immune system.

## Introduction

Plants are constantly exposed to a wide spectrum of microbial pathogens and have evolved both constitutive and inducible defense mechanisms. Constitutive defenses include physical barriers such as waxy epidermal cuticles and cell wall on the plant surface to prevent pathogen penetration (Underwood, 2012). Inducible defenses are activated upon recognition of potential pathogenic microbes by sophisticated surveillance mechanisms in plants (Jones and Dangl, 2006). First, plasma membrane (PM)-localized pattern recognition receptors (PRRs) recognize microbe-associated molecular patterns such as bacterial flagellin and initiate signaling to activate pattern-triggered immunity (PTI), a set of responses including burst of reactive oxygen species (ROS), activation of mitogen-activated protein kinases (MAPKs), and defense gene expression (Jones and Dangl, 2006). Pathogens can secret effectors to plant cells to suppress PTI but some of the effectors may be recognized by plant resistance (R) proteins and activate effector-triggered immunity, a strong defense often manifested as hypersensitive response (HR) (Jones and Dangl, 2006).

**FIGURE 1:**
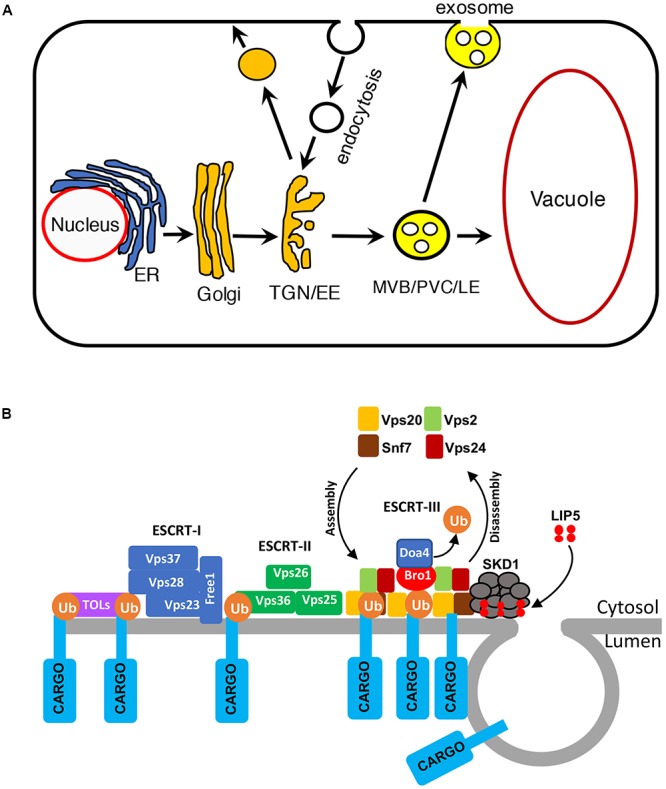
Multivesicular bodies (MVBs) in the endocytic pathway in plants. **(A)** The endocytic pathway in plants. ER, endoplasmic reticulum; TGN, trans-Golgi network; EE, early endosome; MVB, multivesicular body; PVC, prevacuolar compartment; LE, late endosome. **(B)** The ESCRT complexes in MVB biogenesis.

Vesicle trafficking controls the structures of intracellular compartments and communication between cells and environment (Reyes et al., 2011; Paez Valencia et al., 2016). In the endocytic pathway, molecules are delivered from the PM in endocytic vesicles to early endosomes (EEs), which mature into late endosomes (LEs). Multivesicular bodies (MVBs) are LEs that contain intraluminal vesicles (ILVs), which are generated from the invaginations and budding of the limiting membrane into the lumen. Cargo-containing ILVs can be degraded upon fusion with lysosomes/vacuoles or released as exosomes upon fusion with the PM (Hanson and Cashikar, 2012; **Figure 1A**). In plants, MVBs also play important roles in trafficking proteins to the vacuoles in the secretory pathway and are also referred to as prevacuolar compartments (PVCs; Cui et al., 2016; **Figure 1A**). Studies over the past decades have also uncovered critical roles of MVBs in pathogen-triggered trafficking processes important for both the signaling and execution of plant defense. In this review, we discuss the progress in our understanding of the role, action, and regulation of MVB biogenesis and trafficking in plant immunity.

## Pathogen-Induced MVB Biogenesis

Multivesicular bodies mature from trans-Golgi network (TGN)/EEs (Cui et al., 2016; **Figure 1A**). The characteristic ILVs of MVBs originate from the vesicle budding from the limiting membrane into the lumen through the action of protein complexes named ESCRT-0, I, II, and III (endosomal sorting complex required for transport; Wollert and Hurley, 2010; **Figure 1B**). Plants have no canonical ESCRT-0 subunits but contain TOM1-like (TOL) proteins that bind ubiquitin and are required for the endocytosis and vacuolar sorting of the auxin efflux carrier component PIN2 (Korbei et al., 2013; Gao et al., 2017). ESCRT-1 components further cluster ubiquitinated proteins and also recruit ESCRT-II. The Vps25 subunit of ESCRT-II interacts with the Vps20/CHMP6 subunit of ESCRT-III to activates assembly of ESCRT-III on the endosomal membrane for cargo sorting, concentration, and vesicle formation (Wollert and Hurley, 2010). ESCRT-III assembles transiently on the endosome membrane and is then disassembled from the membrane into the cytoplasm after each cycle (Piper and Katzmann, 2007; **Figure 1B**). ESCRT-III disassembly is catalyzed by the Vps4p/SKD1 AAA ATPase, which is activated by Vta1/LIP5 (**Figure 1B**). Both Vps4p/SKD1 and Vta1/LIP5 are critical for MVB biogenesis in yeast and mammalian cells (Yeo et al., 2003; Shiflett et al., 2004; Ward et al., 2005; Azmi et al., 2006). In Arabidopsis, disruption of *SKD1* is lethal, but *lip5* mutants are normal in growth and development (Haas et al., 2007; Wang et al., 2014).

**FIGURE 2:**
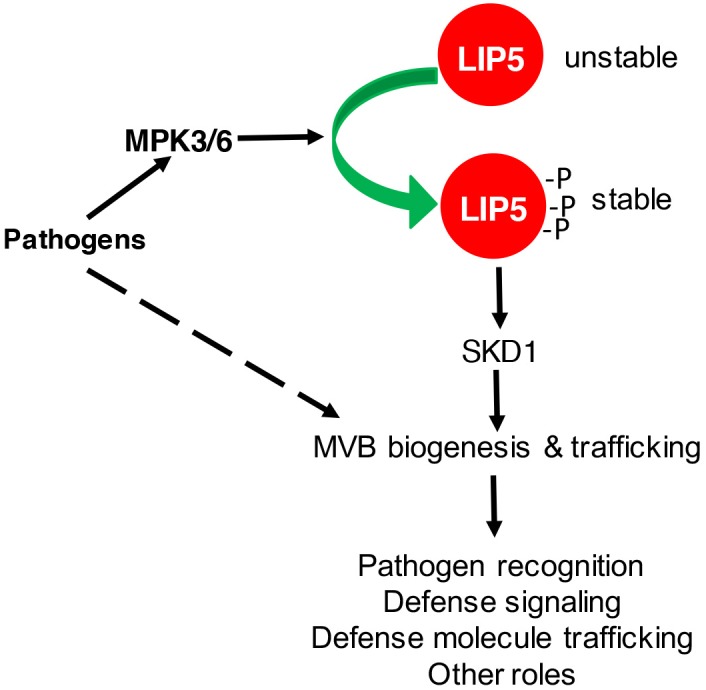
Regulation and role of LIP5 in MVB biogenesis and plant immune responses. Pathogen-responsive MAPK3/6 increases LIP5 stability through phosphorylation. Increased levels of LIP5 stimulate SKD1 and increase MVB biogenesis and trafficking to promote plant immune responses. MVB biogenesis and trafficking could also be stimulated through other unknown mechanisms upon pathogen infection.

Multivesicular body biogenesis and associated endocytosis are pathogen-inducible in plants (An et al., 2006a,b; Wang et al., 2014). After flagellin binding, activated plant PRR FLS2 undergoes rapid endocytosis and accumulates in MVBs before degradation (Robatzek et al., 2006; Beck et al., 2012; Spallek et al., 2013). In the penetration resistance of cereal plants against the powdery mildew fungus, increased formation and relocalization of MVBs were associated with local cell wall appositions (papillae) surrounding the pathogen’s penetration points (An et al., 2006a,b, 2007). Similar relocalization of defense-related molecules such as the PEN3 ATP binding cassette (ABC) transporter possibly through MVBs for cell surface defense has also been found in Arabidopsis (Underwood and Somerville, 2013). Using both the FM1-43 endocytosis marker and the GFP-labeled ARA6 GTPase MVB marker, we have observed that both endocytosis and MVB biogenesis increase in response to *Pseudomonas syringae* in Arabidopsis (Wang et al., 2014). Importantly, pathogen-induced endocytosis and MVB biogenesis was not observed in the *lip5* mutants (Wang et al., 2014). Thus, LIP5 is required for pathogen-induced MVB biogenesis and endocytosis. This role of LIP5 in pathogen-induced MVB biogenesis is dependent on its enhanced protein stability upon phosphorylation by pathogen-responsive MAPK3 and 6 in Arabidopsis (Wang et al., 2014; **Figure 2**).

## MVBs in Defense Signaling

In Arabidopsis, plant receptor FLS2 mediates immunity against bacterial infection through recognition of bacterial flagellin (Gomez-Gomez and Boller, 2000). Following flagellin binding, activated FLS2 undergoes endocytosis and accumulates in MVBs before being degraded in the vacuole (Robatzek et al., 2006; Choi et al., 2013). Several lines of evidence suggest that endocytosis of FLS2 functions as a molecular mechanism not only for its attenuation but also for proper immune responses (Salomon and Robatzek, 2006). First, mutant FLS2 proteins impaired in endocytosis are compromised in conferring flagellin-triggered responses. FLS2^T867V^, which carries a substitution of the potentially phosphorylated residue threonine-867, had impaired endocytosis and could not complement the enhanced disease susceptibility in the *fls2* mutants (Robatzek et al., 2006). Endocytosis of FLS2^P1076A^, which contains a mutation in a PEST motif, was abolished, and the mutant FLS2 protein failed to confer flagellin-induced ROS production in the *fls2* mutants (Robatzek et al., 2006). Second, treatment with vesicular trafficking inhibitors such as wortmannin-reduced FLS2 endocytosis and impaired flagellin-induced production of ROS (Smith et al., 2014). Third, endocytosis and trafficking of FLS2 and other PRRs require clathrin and clathrin-dependent endocytosis is involved in FLS2-mediated stomatal closure and callose deposition (Mbengue et al., 2016). In addition, endocytosed FLS2 associates and co-localizes with the VPS37-1 and VPS28-2 components of ESCRT-I (Spallek et al., 2013). In Arabidopsis *vps37-1* and *vps28-2* mutants, FLS2 endocytosis was reduced and MVB sorting was compromised (Spallek et al., 2013). When surface-inoculated with virulent *P. syringae*, *vps37-1*, and *vps28-2* also supported increased bacterial growth due to compromised stomatal closure, even though ROS production and MAPK activation were normal in the mutants (Spallek et al., 2013). Finally, flagellin peptide flg22 could move to distal organs through vascular tissues (Jelenska et al., 2017). Delivery of flg22 to vascular tissues and its long-distance transport were dependent on its endocytosis together with the FLS2 receptor (Jelenska et al., 2017). Thus, FLS2 endocytosis is also required for long-distance movement of flg22, possibly for systemic immune responses.

Multivesicular bodies also play a role in the functioning of NB-LRR R proteins. Potato R protein R3a can mount an immune response upon recognition of the Avr3a^K80I103^ (Avr3a^KI^) effector from *Phytophthora infestans*, but not the Avr3a^E80M103^ (Avr3a^EM^) effector, which differs from Avr3a^EM^ in only two amino acids (Engelhardt et al., 2012). R3a is normally in the cytoplasm but relocalized to MVBs when co-expressed with its cognate effector Avr3a^KI^, but not with Avr3a^EM^ (Engelhardt et al., 2012). Co-expressed Avr3a^KI^, but not Avr3a^EM^, is also relocalized to MVBs in a R3a-dependent manner prior to R3a-mediated HR. Relocalization of R3a to MVBs is inhibited by vesicle trafficking inhibitors brefeldin and wortmannin, which also blocked R3a/Avr3a^KI^-mediated HR, indicating that relocalization of R3a is required for the immune response (Engelhardt et al., 2012). Thus, MVBs act as subcellular compartments from which signaling can be initiated from internalized PRRs and internal R proteins to activate immune responses. Studies in other organisms also indicate MVBs can function in signaling or even as signaling organelles. During Wnt signal transduction in animals, Wnt signaling can trigger sequestration of glycogen synthase kinase into MVBs, allowing the activation of many cytosolic proteins (Dobrowolski and De Robertis, 2011). In addition, MVBs containing nerve growth factor (NGF) and its receptor TrkA in mouse sympathetic neurons can evade lysosomal fusion and instead evolve into membrane vesicles that are signaling components (Ye et al., 2018).

## MVBs in Cell Surface Defense

Trafficking defense-related molecules through MVBs plays a critical role for defense against invading pathogens at plant cell surface. In the interactions between plants and filamentous pathogens, the spores of the pathogens germinate on the leaf surface and develop infection pegs to invade the epidermal cells, which can induce a range of plant defense responses including the formation of local cell wall appositions (papilla) at the sites of pathogen attack (Collins et al., 2003; Assaad et al., 2004; Schulze-Lefert, 2004). Studies have showed accumulation of MVBs and cell wall-associated paramural bodies (PMBs) in the vicinity of pathogen-induced papillae (An et al., 2006a,b, 2007). PMBs, which are situated between the cell wall and the PM, likely result from the fusion of MVBs with the PM (Marchant and Robards, 1968). Plant MVBs and PMBs have been observed near papillae in plant cells infected by pathogenic fungal, bacteria, and nematodes for delivery of defense-related molecules including phytoalexins, callose, and ROS to papillae (An et al., 2006a,b; Meyer et al., 2009; Bohlenius et al., 2010; Nielsen et al., 2012). In Arabidopsis, two pathways have been identified in the penetration resistance to the non-adapted powdery mildew fungus *Blumeria graminis* f. sp hordei (Bgh) defined by the PM syntaxin, PEN1, and the β-glucoside hydrolase PEN2. The PEN1-mediated pathway delivers building materials for the papilla, while PEN2 functions with the PEN3 ABC transporter in mediating the synthesis and export of antimicrobial metabolites to the attack sites. Relocalization of defense-related molecules such as PEN1 and PEN3 for cell surface defense in response to conserved pathogen elicitors has also been observed in Arabidopsis (Underwood and Somerville, 2013). Given the diverse roles in recycling, degradation, and relocalization of defense-related molecules and different structural appearances of MVBs and associated PMBs, it would be of interest to determine whether there exist multiple pathways for MVB biogenesis and trafficking and whether ESCRT complexes are involved in the formation of PMBs.

Upon successful penetration, filamentous pathogens can develop a special feeding structure into plant cells called haustoria. Each haustorium is surrounded by the PM of the plant cell termed extrahaustorial membrane (EHM), which is likely synthesized *de novo* (Berkey et al., 2017). In tobacco (*Nicotiana benthamiana*) cells invaded by oomycete pathogen *P. infestans*, the Rab7 GTPase RabG3c MVB marker protein, but not a tonoplast-localized sucrose transporter, is recruited to the EHM (Bozkurt et al., 2015). In Arabidopsis, the Rab5 GTPase, also an MVB marker, accumulates in the EHM after infection with a powdery mildew fungus (Nielsen et al., 2017). Thus, specific rerouting of MVBs from the vacuole to the host–pathogen interface may participate in the formation or modulation of the EHM. Importantly, cell surface immune receptors such as FLS2 and RPW8 resistance proteins are also recruited to the EHM upon pathogen infection, probably as a host border control mechanism at the plant–pathogen interface (Eckardt, 2009; Kim et al., 2014; Bozkurt et al., 2015; Berkey et al., 2017).

Further evidence for a role of plant MVBs in plant immune responses has been provided from plant extracellular vesicles (EVs), which are likely generated from the fusion of MVBs with the PM. EVs from Arabidopsis rosettes contain PEN1, RIN4 (RPM1-INTERACTING PROTEIN4), RIN4-interacting proteins and proteins involved in metabolism and transport of antimicrobial compounds (Rutter and Innes, 2017). EV secretion is enhanced in Arabidopsis after *P. syringae* infection (Rutter and Innes, 2017). Comparison of EV proteins with other published proteomes indicates that they are most similar to those from the TGN/MVB compartments (Rutter and Innes, 2017), supporting that EVs are derived from the ILVs of MVBs. EVs isolated from the extracellular fluids of sunflower seedlings are also enriched in defense proteins and could be taken up by the phytopathogenic fungus *Sclerotinia sclerotiorum* to cause growth inhibition, morphological changes, and cell death (Regente et al., 2017). These studies provide strong evidence for the existence of plant exosomes and their antimicrobial roles in plant immune responses.

## Genetic Evidence for the Role of MVBs in Plant Immunity

In spite of the extensive microscopic data, genetic analysis of the role of MVBs in plant immune system is relatively limited, in part, because of the lethal phenotype of mutants for key components of MVB biogenesis such as SKD1 (Haas et al., 2007; Spitzer et al., 2009). However, some components required for MVB biogenesis and trafficking are encoded by gene families and disruption of individual family members can allow for genetic analysis of their specific roles in plant defense. For example, genetic analysis of the VPS37-1 and VPS28-2 components of the ESCRT-I complex has revealed their role in FLS2 endocytosis and stomata-mediated defense (Spallek et al., 2013). The barley ARF GTPase ARFA1b/1c has been localized to MVBs and shown to be important for callose-deposition in papillae and penetration resistance of barley (Bohlenius et al., 2010). Furthermore, an ARF-GTP exchange factor, GNOM, is involved in papilla formation, callose deposition, and penetration resistance (Nielsen et al., 2012). However, the MVB localization of the ARF1 factor was later disputed, and evidence was presented for its localization to the Golgi and TGN (Robinson et al., 2011). More recently, it has been reported that endosome-associated VPS9a from Arabidopsis, the conserved guanine-nucleotide exchange factor activating Rab5 GTPases required for MVB maturation and fusion with other membranes, plays a critical role in both preinvasive and, to a greater extent, post-invasive immunity against a non-adapted powdery mildew fungus, as well as defense against an adapted powdery mildew fungus (Nielsen et al., 2017).

To analyze LIP5-mediated MVB biogenesis in plant immune responses, we have characterized two independent *lip5* mutants. Both *lip5* mutants are normal in growth and development but are highly susceptible to *P. syringae* (Wang et al., 2014). The critical role of LIP5 in plant immunity is dependent on its interaction with SKD1, indicating that the role of LIP5 in plant immune system is mediated by its action in MVB biogenesis. Basal levels of endocytosis and MVB biogenesis under normal conditions were normal in the *lip5* mutants, consistent with its normal growth and development. After pathogen infection, both the endocytosis and MVB biogenesis were induced in a LIP5-dependent manner (Wang et al., 2014). Pathogen infection resulted in increased numbers of MVBs and PMBs in wild-type plants but not in the *lip5* mutants (Wang et al., 2014). Therefore, pathogen-responsive MVB biogenesis and associated trafficking are activated by pathogen-responsive MAPK3/6 through LIP5 phosphorylation and plays an important positive role in plant immune system, most likely through trafficking and deployment of defense-related molecules at the cell surface to counter invading pathogens (**Figure 2**).

Intriguingly, a recent study on a rice AAA ATPase, LRD6-6, has indicated a negative role of MVB-mediated vesicular trafficking in plant immunity (Zhu et al., 2016). Rice LRD6-6 was identified from a rice lesion resembling disease (*lrd*) mutant resulting from insertion of a 534-nt DNA fragment in the protein-coding sequence of LRD6-6, which would disrupt its function (Zhu et al., 2016). The *lrd6-6* mutant displays spontaneous lesions, enhanced disease resistance, and increased accumulation of antimicrobial compounds (Zhu et al., 2016). LRD6-6 is structurally related to SKD1, interacted with components of ESCRT-III, and associated with MVBs (Zhu et al., 2016). In addition, the *lrd6-6* mutant was altered in expression of genes associated with MVB-mediated trafficking and defective in the trafficking of the Arabidopsis soluble vacuolar carboxypeptidase Y (AtCPY) from the ER to the vacuole, which is mediated by the secretory pathway through MVBs/PVCs (Zhu et al., 2016). However, sequence analysis reveals that Arabidopsis SKD1 protein is mostly closely related to three other proteins encoded by LOC_Os01g04814, LOC_Os02g06490, LOC_Os01g04840 in rice (unpublished data). On the other hand, rice LRD6-6 is most similar to the spastin protein, an AAA ATPase widely found in eukaryotes with a microtubule-severing activity associated with membrane trafficking, assembly of nuclear protein complexes, cytokinesis, and secretion (Connell et al., 2009; Lumb et al., 2012). Furthermore, the genes with altered expression in *lrd6-6* are mostly associated with early stages of the secretory pathway encoding membrane coat, clathrin coat of coated pit, ER membrane, and clathrin coat of TGN vesicle (Zhu et al., 2016), which could explain its defect in the transport of the Arabidopsis AtCPY from the ER to the vacuole (Zhu et al., 2016). Plant MVBs, also known as PVCs, play important roles in mediating protein traffic in both the endocytic and secretory pathways (Cui et al., 2016). Disruption of the secretory pathway could lead to impaired traffic of vacuolar cargo, defects in vacuolar biogenesis, or even cell death as observed in the *ldr6-6* mutant.

## Conclusion and Future Prospects

Important progress has been made in identifying components important for plant immunity by participating in pathogen-responsive MVB biogenesis and associated trafficking for relocalization of cellular components important for effector recognition, modulation of PRR activity, delivery of defense-related proteins, and antimicrobial metabolites, building of encasement surrounding invading pathogens and regulation of cell growth and death. Over the next few years, it is expected that additional components in pathogen-responsive MVB and associated trafficking will be identified and their roles analyzed. It has become increasingly apparent that the timing and destinations of endosomal trafficking through MVBs are highly cargo-specific, and it is important to elucidate the underlying mechanisms. Further analysis will also be necessary to comprehensively establish the specific activities of the exosome content including possible regulatory activities in communication among plant cells or between plant and pathogen cells (Rutter and Innes, 2018). These studies can lead to a better understanding of plant endosomal trafficking and plant immune responses.

## Author Contributions

CZ and ZC conceived the idea. XL, HB, and ZC wrote the manuscript. XL, HB, ZW, MW, BF, CZ, and ZC evaluated the manuscript. All authors read and approved the manuscript.

## Conflict of Interest Statement

The authors declare that the research was conducted in the absence of any commercial or financial relationships that could be construed as a potential conflict of interest.
